# Current insights in non-invasive ventilation for the treatment of neonatal respiratory disease

**DOI:** 10.1186/s13052-019-0707-x

**Published:** 2019-08-19

**Authors:** Dhivya Lakshmi Permall, Asfia Banu Pasha, Xiao-qing Chen

**Affiliations:** Department of Pediatrics, The First Affiliated Hospital of Nanjing Medical University, No 300, Guangzhou Road, Nanjing, Jiangsu China

**Keywords:** Newborn, Non-invasive ventilation, Continuous positive airway pressure, Nasal intermittent positive pressure ventilation, Respiratory distress syndrome

## Abstract

Deleterious consequences of the management of respiratory distress syndrome (RDS) with invasive ventilation have led to more in-depth investigation of non-invasive ventilation (NIV) modalities. NIV has significantly and positively altered the treatment outcomes and improved mortality rates of preterm infants with RDS. Among the different NIV modes, nasal intermittent positive pressure ventilation (NIPPV) has shown considerable benefits compared to nasal continuous positive airway pressure (NCPAP). Despite reports of heated humidified high-flow nasal cannula’s (HHHFNC) non-inferiority compared to NCPAP, some trials have been terminated due to high treatment failure rates with HHHFNC use. Moreover, RDS management with the combination of INSURE (INtubation SURfactant Extubation) technique and NIV ensures higher success rates. This review elaborates on the currently used various modes of NIV and novel techniques are also briefly discussed.

## Introduction

Renewed interest was sparked in NIV modes due to rising incidence of bronchopulmonary dysplasia (BPD) with the use of mechanical ventilation. Despite its benefits in terms of survival of preterm infants, invasive mechanical ventilation for the treatment of neonatal respiratory disease has also elicited an increase in the number of BPD sufferers [1]. The outcomes of BPD are multiple and cause long-term respiratory and neurologic consequences for the patient, leading to a poor quality of life, with increased fatality risk [2, 3]. Cerebral palsy, movement disorders, abnormal motor skill development and visual and auditory disorders are other reported consequences of BPD [4]. The current goal is to find the best NIV technique and its optimal settings for respiratory support in RDS management for the different groups of preterm infants.

## Modes of non-invasive ventilation

### Nasal continuous positive airway pressure (NCPAP)

NCPAP is the most widely used non-invasive ventilation mode in neonatal intensive care units (NICUs) [5]. The basis of NCPAP is keeping the airways open and maintaining functional residual capacity (FRC) [6]. The mechanism of action comprises an increase in the pharyngeal cross-sectional area, enhancement of diaphragmatic activity, improved pulmonary compliance, and decreased airway resistance which leads to less work of breathing, decreased incidence of apnea and better ventilation-perfusion [6, 7]. Newer NCPAP interfaces such as nasal masks, single or bi-nasal prongs have now replaced the older interface models [6]. Chen et al. [5] proposed a new strategy to improve the quality of NCPAP delivery in the NICU. NCPAP kits with a mobile cart and written nursing protocols were used in the NICU to decrease the NCPAP set time, patient discomfort and complications associated with NCPAP. Another aim of this project was to provide the same standard of nursing care to all the patients.

### Nasal intermittent positive pressure ventilation (NIPPV)

Several modes of NIPPV have been described in literature, namely nasal intermittent mandatory ventilation (NIMV), non-invasive pressure support ventilation, and bi-level CPAP [8]. It can be further classified as synchronized (patient-triggered) NIPPV (SNIPPV) and non-synchronized (machine-triggered) NIPPV (NS-NIPPV) [8]. Application of NIPPV combines NCPAP with additional intermittent breaths above the baseline and the modifiable parameters are positive end expiratory pressure (PEEP), peak inspiratory pressure (PIP), respiratory rate and inspiratory time (Ti) [9–11]. The periodic breaths increase tidal volume leading to enhanced removal of CO2, sustained alveolar ventilation during episodes of apnea and increased FRC [8, 9]. The efficacy of NIPPV is enhanced by the combined usage of early surfactant use in RDS [12]. This mode of NIV has a greater ability to reduce apneic and bradycardic episodes in preterm infants compared to NCPAP [13]. While alterations in pressure and lung volume are not considered to be the actions of NIPPV, proposed mechanisms are: pressure delivery to lower airways, alveoli micro-recruitment, pharyngeal inflation and elicitation of an increased inspiratory reflex (Head’s paradoxical reflex) [1, 9]. Even with the prevalence of NIPPV worldwide, the types of devices used and the mode of delivery vary among countries [9]. Most ventilators can be used to provide NS-NIPPV but SNIPPV can only be generated by Infant Flow SiPAP and Infant Flow Advance, since the Infant Star ventilator is now unavailable [11]. For synchronization, the most frequently used device is the Graseby capsule (GC), which is placed in the subxiphoid area to track the respiratory effort [9, 14]. Most studies/units use the short bi-nasal prongs as the interface for NIPPV, although, use of masks and long nasopharyngeal tubes have been reported [1, 11]. The popularity of NIPPV is rising since its comparison to NCPAP has demonstrated significant decrease in respiratory failure, re-intubation rates and extubation failure [15]. However, in one of the largest studies by Kirpalani et al. [16], NIPPV did not prove to be superior to CPAP for extremely low birth weight (ELBW) infants born before 30 weeks of gestation for outcomes such as survival with BPD or death.

### **B**i-level nasal CPAP (BiPAP)

BiPAP has a mechanism similar to NIPPV, and it is usually included within the broad term of NIPPV [9]. It provides cycles of alternating high and low levels of positive airway pressure at preset intervals of time, not synchronous to the infant’s breathing pattern [9, 15]. The pressure delivered by BiPAP is lower than NIPPV and the higher and lower positive airway pressure levels differ by no more than 3–4 cmH2O [1, 17]. Also, with BiPAP, the Ti is longer and cycle rate is lower [1]. Limited tools are available for BiPAP delivery to neonates [9].

### High flow nasal cannula (HFNC)

The latest addition to the NIV family in the NICU is the HHHFNC, which delivers heated and humidified gas through the usage of the HFNC system [18]. The HFNC system consists of small-sized, bi-nasal prongs that do not occlude the nostrils, through which oxygen or a mixture of oxygen and air is delivered at a flow rate of > 1 L/min [19] or > 2 L/min [11]. Preconditioning of gases to mimic the normal upper airway conditions is a crucial characteristic which helps to diminish energy consumption of the body, to avoid proximal airway mucosal dryness and injury [1, 18]. Moreover, although not proven, the mechanisms of action of HHHFNC are thought to include: (1) decreased airway resistance and work of breathing, (2) increase the efficiency of gas exchange by the washout of nasopharyngeal dead space in the upper respiratory tract, and (3) supply of positive distending pressure [1, 18, 20]. Decreased rates of nasal trauma and infant pain scores have been revealed with the use of HHHFNC [21]. Despite the uncertain safety of HHHFNC, surveys demonstrate its increasing use in about two-thirds of NICUs in developed countries, such as the United States, Australia and New Zealand [21]. Its rising popularity is mainly due to its ease of application and maintenance, thus being the preferred NIV mode of physicians and nurses [18, 19]. The fact that there is no sealing required also causes less distress to the infants [19]. In spite of the numerous benefits imparted by HHHFNC, the major concern related to this NIV mode is the unavailability of monitoring the pressure it delivers [22].

## Comparison

### NCPAP vs NIPPV

In a meta-analysis of 10 trials, with 1061 preterm infants requiring respiratory support for respiratory distress disease, NIPPV proved to be more efficient than NCPAP for the prevention of respiratory failure and for reducing need for intubation [23]. Among the 10 trials, only Ramanathan et al. [24] showed a decrease in BPD incidence and it has been attributed to early surfactant administration prior to the use of respiratory support. Early use of NIPPV instead of NCPAP for preterm RDS patients showed lesser need for mechanical ventilation by 72 h of age and by 7 days of age [25, 26]. Tang et al. [27] also found NIPPV to reduce intubation requirement, with a slight decrease in BPD incidence and increase in extubation success. Similarly, Yuan et al. [28] found less intubation in preterm infants supported by NIPPV. NIMV compared to NCPAP as initial treatment for RDS in preterm newborns of < 35 weeks of gestation demonstrated decreased need for intubation and decreased BPD rate with NIMV [29]. Silveira et al. [30] found that for preterm infants of gestational age < 37 weeks and birth weight < 2500 g, failure on NIPPV support compared to CPAP was less likely and the rate of intubation was higher when using CPAP. Moreover, occurrence of apnea episodes was lower in the NIPPV group. The significant effect of apneic episodes reduction with NIPPV compared to NCPAP has been reported by several studies [13, 27, 31, 32]. In a review evaluating the use of NIPPV and NCPAP as post-extubation methods, the results were statistically significant in showing stronger effect of NIPPV in reducing post-extubation failure [33]. Furthermore, synchronized NIMV has proven to be efficient in improving extubation success in very low birth weight (VLBW) infants in the first 72 h post-extubation [32]. As Ramanathan et al. [24] have shown the beneficial association of INSURE followed by NIPPV on BPD, additionally, another RCT [34] compared the use of NIPPV and NCPAP after the INSURE approach in premature infants of < 34 weeks of gestation suffering from RDS in terms of efficacy and complications of the two NIV modes. It revealed significantly lower re-intubation rates, reduced length of hospitalization and decreased BPD rates in the NIPPV group. Oncel et al. [35] compared NCPAP and NIPPV as the primary mode of respiratory support within the minimally invasive surfactant therapy (MIST) for 200 preterm infants with respiratory distress not requiring intubation. They showed the diminished requirement of surfactant and invasive ventilation in the NIPPV group, but no effect on BPD outcome. Li et al. [36] found a significant decrease in the need for intubation in the subset of infants who received surfactant before NIPPV, confirming the beneficial effect of early surfactant therapy. Salvo et al. [37] retrospectively compared NCPAP, SNIPPV and nasal BiPAP to assess their efficiency as initial treatment for RDS in VLBW infants. They found a significantly higher frequency of NIV failure within the first 5 days of life in the NCPAP group as compared to SNIPPV and BiPAP groups, depicting the benefits of using SNIPPV or BiPAP as primary treatment for VLBW infants with RDS. Moreover, there was no difference in the SNIPPV and BiPAP groups.

### NCPAP vs BiPAP

According to a small study by Lista et al. [38], BiPAP was superior to NCPAP in infants with moderate RDS between 28 and 34 weeks’ gestational age. Although similar serum cytokine levels were observed in both groups, reduced respiratory support, supplemental oxygen and hospital stay were advantageous outcomes seen with BiPAP support. Furthermore, Rong et al. [39] found BiPAP to be more effective than NCPAP in reducing the intubation requirement in the first 72 h of life for infants of ≤32 weeks’ gestational age but BiPAP did not modify the BPD incidence. The use of BiPAP has also demonstrated improvement in gas exchange compared to NCPAP [40]. In comparing nasal BiPAP to NCPAP as post-extubation support in 540 preterm infants, Victor et al. [41] found no additional benefit with nasal BiPAP as post-extubation support.

### NCPAP vs HHHFNC

A large RCT involving 432 preterm infants found no difference in terms of efficacy and safety of HHHFNC compared to NCPAP, whether as initial respiratory support or as post-extubation support [42]. Accordingly, the authors support the non-inferiority of HHHFNC when compared to NCPAP. However, the rate of nasal trauma was significant in the NCPAP group. The large HIPSTER trial [21] designed to compare HFNC to NCPAP as early respiratory support for infants with respiratory distress without the use of surfactant, was interrupted since the treatment failure rate was significantly higher in the HFNC group. Nonetheless, a significantly higher frequency of nasal trauma and pulmonary air leaks was observed with NCPAP. One of the recent trials comparing HFNC to NCPAP was also interrupted due to significantly higher treatment failure rate in the HFNC group [43]. Since high flow therapy fairs better as post-extubation support, surfactant administration might be the key to the success of high flow therapy. In addition, whether high-flow therapy is used as primary or post-extubation support in preterm infants, rescue NCPAP should be available in case of high-flow therapy failure to avoid intubation [20]. Another small RCT with 54 preterm infants with RDS randomised to HFNC or NCPAP as post extubation support after INSURE approach observed an increase rate of re-intubation in the HFNC group compared with the NCPAP group [44]. However, the authors emphasized that the use of higher flow rates of > 4 L/min might resolve this problem. Lavizzari et al. [45] evaluated the efficacy of HHHFNC when compared to NCPAP or BiPAP as the initial treatment for mild to moderate RDS in preterm neonates of > 28 weeks’ gestational age. HHHFNC and NCPAP/BiPAP displayed similar efficacy with regard to the requirement of intubation within 72 h since the start of respiratory support. The results of the currently ongoing HUNTER trial in Australia, comparing HHHFNC to NCPAP as primary support in preterm infants with RDS, is awaited to determine if HHHFNC is consistently non-inferior to NCPAP as primary respiratory support [46].

### NIPPV vs BIPAP

In comparing SNIPPV and BiPAP, Salvo and al [47]. concluded that both NIV strategies are valuable in the treatment of early RDS in VLBW neonates. In the study comparing NCPAP, SNIPPV and BiPAP as initial treatment for RDS in VLBW infants, the efficacies of SNIPPV and BiPAP were also similar [48].

### NIPPV vs HHHFNC

A pilot study conducted to compare HHHFNC to NIPPV as the primary therapy for RDS revealed comparable use of both methods as initial treatment for RDS and in terms of preventing intubation in infants < 35 weeks’ gestation and birth weight > 1000 g [49]. However, further larger trials are warranted before initiating the use of HHHFNC as a primary treatment for neonatal respiratory disease.

### HHHFNC vs BIPAP

The only study to compare HHHFNC and NCPAP/BiPAP was conducted by Lavizzari et al. [45], showing HHHFNC to have similar efficacy to both NCPAP and BiPAP as the initial mode of NIV support in preterm neonates of > 29 weeks’ gestation with mild-moderate RDS.

## Synchronised or not

One of the first studies to demonstrate work of breathing reduction in preterm infants with the use of SNIPPV was done more than 10 years ago [50]. Chang et al. [51] also reported reduced inspiratory effort when using synchronized NIMV and Huang et al. [52] supported these benefits of synchronized ventilation. Other reported advantageous aspects of SNIPPV include improved thoraco-abdominal synchrony, reduced need of intubation and lower incidences of desaturations, bradycardias and central apnea [9, 33, 53]. Decrease in BPD and air leakage was also noted with SNIPPV [33]. The use of SNIPPV on 78 infants of < 32 weeks of gestation as post-extubation support or after NCPAP failure, showed a reduced need for intubation in 74.4% of these preterm infants with respiratory failure [54]. Khalaf et al. [55] demonstrated superiority of SNIPPV over NCPAP for extubation success in RDS patients ≤34 weeks’ gestational age. SNIPPV has also shown potential as a favorable mode of respiratory support after the INSURE approach since it decreases the need for mechanical ventilation and limits the requirement of additional surfactant doses. It is thought to enhance the distribution of surfactant in the lungs [56]. Comparison of SNIPPV with BiPAP revealed similar efficacy of both methods [48]. One of the flaws of the SiPAP system remains its inability to respond to all detected breaths at higher breath rates, thus lower peak pressures are delivered as compared with the previously used GC with the Infant Star ventilator [9, 57, 58].

## Non-invasive ventilation in the delivery room and NICU

The only currently used NIV mode in the delivery room or for stabilization in the first few hours of life is NCPAP, either used alone or with the INSURE technique, thus requiring a brief duration of intubation [59, 60]. In comparing early NCPAP to intubation, Morley et al. [61] found no significant decrease in BPD or mortality between the two study groups. The SUPPORT trial [62] compared early CPAP treatment with early surfactant treatment and mechanical ventilation in extremely preterm infants started in the delivery room and although no significant difference was noted in the mortality or BPD rates, the CPAP group resulted in decreased intubation rate, decreased use of postnatal corticosteroid and reduced ventilation time. However, initial application of NCPAP followed by selective surfactant use in extremely preterm infants can decrease the incidence of BPD or mortality rates [63]. A Cochrane review also found decrease incidence of BPD, lesser need for intubation and lesser occurrence of air leak syndromes in infants at risk of or with RDS, treated with early surfactant followed by NCPAP [64]. According to the analysis of four RCTs, one extra infant could survive to 36 weeks without BPD for every 25 babies treated with NCPAP in the delivery room instead of being intubated [65]. Despite the overall decreased risk of BPD with early NCPAP use in the delivery room, NCPAP still has a high failure rate, with a 50% failure in VLBW infants reported [59, 66]. The risk factors of NCPAP failure are infants with smaller gestational age, male gender, low birth weight infants, FiO2 > 0.25 at 1 and 2 h of age [67, 68]. The cause of NCPAP failure in premature infants is often RDS and it can be predicted by FiO2 ≥ 0.3 in the first hours of life [69]. The timing of surfactant is a key factor for BPD prevention as administration > 2 h after birth, known as late rescue surfactant treatment, has shown decreased efficiency in reducing BPD [68]. Knowing the high-risk group of preterm neonates prone to NCPAP failure might improve the timing of surfactant administration and avoid unnecessary NCPAP therapy [67]. This high NCPAP failure rate finding has led to the use of sustained lung inflation (SLI), which is the delivery of a high peak pressure of 20–25 cmH2O for a duration of 10–15 s using a face mask or nasopharyngeal tube [66]. SLI combined with NCPAP instead of NCPAP alone in the delivery room revealed a reduced need for invasive ventilation in the initial 72 h of life for infants at high risk of RDS [70]. However, no change in the incidence of BPD was observed with SLI use [70]. The ongoing SAIL (Sustained Aeration of Infant Lungs) trial is focused on evaluating the effect of sustained inflation versus standard positive pressure ventilation [71].

## Newer NIV modes

Nasal high-frequency oscillation ventilation (nHFOV), provides an oscillatory pressure waveform to the airways, without synchrony with the infant’s breath, aiding to enhance CO2 elimination and alveolar recruitment [72, 73]. A study using a term-newborn model showed the superiority of nHFOV in terms of CO2 elimination compared to NCPAP and NIPPV, with thrice the effect of NIPPV [74]. Additionally, it has been reported to significantly decrease CO2 levels, desaturations and frequency of apnea and bradycardia episodes [75]. The frequent adverse effects observed with nHFOV are upper airway obstruction due to increased secretions, thick, viscous secretions and abdominal distention [72, 76]. The formation of extremely viscous secretions in the upper airway has been attributed to usage of low nHFOV frequencies with high amplitudes [73]. A recent study compared nHFOV to NCPAP in preterm infants (28–34 weeks) with moderate to severe respiratory distress post-INSURE [77]. They found a significant decrease for intubation requirement when using nHFOV. Further studies are required to assess and compare various devices and interfaces to deliver nHFOV and to compare nHFOV to the more commonly used NIV techniques.

Neurally adjusted ventilatory assist (NAVA) can be provided invasively and non-invasively in spontaneously breathing infants. It is patient-controlled and utilizes diaphragm electrical activity (Edi) to deliver synchronised, pressure-controlled breaths via a ventilator [78]. Central apnea, indicated by the lack of Edi signal, can trigger the back-up ventilation mode of the NAVA system. Since this would resolve the issue of NCPAP failure due to apneic episodes, NAVA would be an ideal alternative method to deliver NCPAP. Moreover, the synchrony achieved using NAVA can allow for earlier extubation [78]. A clinical guideline for the use of NAVA in neonates, by Stein et al., defined NIV-NAVA to be similar to invasive NAVA but ventilation mode delivery is via nasal prongs or single nasal-pharyngeal tube or a mask. NIV-NAVA comprises a leak compensation system which applies to leaks as high as 95%. The benefits of both NAVA and NIV-NAVA are similar, namely better patient-ventilator interaction and synchrony and improved gas exchange efficiency [78, 79]. The ease of use of NIV-NAVA will undoubtedly promote its growing use in NICUs worldwide.

## Conclusion

In the search for the optimal NIV approach for successful respiratory support in RDS management and BPD prevention, further research and study is still called for. NIPPV is rapidly replacing NCPAP due to its remarkable benefits. NAVA, nHFOV and SNIPPV are promising interventions but they require larger RCTs to confirm their safety and efficacy in various infant groups as compared to more familiar NIV modes. Although the long-term outcomes of NIV-NAVA are still to be determined, it is potentially one of the NIV modes that may surpass the standard respiratory support strategies in the near future.

## Data Availability

Not applicable.

## Abbreviations

BiPAP: **B**i-level nasal CPAP
BPD: Bronchopulmonary dysplasia
ELBW: Extremely low birth weight
FRC: Functional residual capacity
GC: Graseby capsule
HFNC: High flow nasal cannula
HHHFNC: Heated humidified high-flow nasal cannula
NAVA: Neurally adjusted ventilatory assist
NCPAP: Nasal continuous positive airway pressure
nHFOV: Nasal high-frequency oscillation ventilation
NICU: Neonatal intensive care unit
NIMV: Nasal intermittent mandatory ventilation
NIPPV: Nasal intermittent positive pressure ventilation
NIV: Non-invasive ventilation
NS-NIPPV: Non-synchronized nasal intermittent positive pressure ventilation
PEEP: Positive end expiratory pressure
PIP: Peak inspiratory pressure
RDS: Respiratory distress syndrome
SLI: Sustained lung inflation
SNIPPV: Synchronized nasal intermittent positive pressure ventilation
Ti: Inspiratory time
VLBW: Very low birth weight
